# Colitis in a patient with familial Mediterranean fever: Is it Crohn's disease or ulcerative colitis?

**DOI:** 10.1002/deo2.70013

**Published:** 2024-09-18

**Authors:** Ayano Hoshi, Yuichi Shimodate, Tatsuhiro Gotoda, Rio Takezawa, Naoyuki Nishimura, Hirokazu Mouri, Kazuhiro Matsueda, Motowo Mizuno, Takayuki Matsumoto

**Affiliations:** ^1^ Department of Gastroenterology and Hepatology Kurashiki Central Hospital Okayama Japan; ^2^ Department of Internal Medicine Division of Gastroenterology and Hepatology Iwate Medical University School of Medicine Iwate Japan

**Keywords:** colitis, Crohn's disease, familial Mediterranean fever, inflammatory bowel disease, ulcerative colitis

## Abstract

A 24‐year‐old woman was referred to our hospital with joint pain, fever, abdominal pain, and diarrhea. A colonoscopy revealed longitudinal ulcers with a cobblestone appearance throughout the entire colon, suggestive of Crohn's disease. However, treatment with 5‐aminosalicylic acid, azathioprine, and infliximab failed to achieve clinical remission. A colonoscopy 5 months later revealed a diffusely spreading granular mucosa without visible vasculature, compatible with active ulcerative colitis. Based on these serial changes in colonic lesions, we tested the patient for *MEFV* gene mutations and found variants E148Q and L110P in exon 2. Administration of colchicine resulted in complete clinical remission. Our experience suggests that drastic changes in the features of colonic inflammation may be a clue to the diagnosis of enterocolitis associated with familial Mediterranean fever.

## INTRODUCTION

It is widely recognized that patients with familial Mediterranean fever (FMF) experience inflammatory intestinal lesions that can resemble those of ulcerative colitis (UC) or Crohn's disease (CD).^1^, ^2^, ^3^, ^4^, ^5^ However, inflammatory intestinal lesions specific to FMF have not been characterized to date. We report our experience with a patient who had proctocolitis occurring in the setting of FMF. Serial colonoscopy revealed drastic changes, from an appearance consistent with CD to one consistent with UC, over a period of 10 months.

## CASE REPORT

A 24‐year‐old woman was referred to our hospital with joint pain, fever, abdominal pain, polyarthralgia predominantly in the shoulder and the hip joints, and five episodes of diarrhea per day for 2 months. The symptoms would disappear after several days, and a similar cycle repeated every few weeks. She had a history of hemorrhoid surgery 3 years prior. She did not have a family history of inflammatory bowel disease (IBD) or FMF. Laboratory tests at the time of admission revealed markedly elevated serum C‐reactive protein (CRP) and amyloid A protein, hypoalbuminemia, slight anemia, and thrombocytosis (Table 1). Her stool and blood were negative for any pathogens.

**TABLE 1:** Laboratory data on admission.

RBC	4.16 × 10^6^/µL	TP	7.6 g/dL	Na	138 mmol/L
Hb	10.4 g/dL	Alb	2.9 g/dL	K	4.4 mmol/L
WBC	7000/µL	T‐BIL	0.6 mg/dL	Cl	103 mmol/L
PLT	54.2 × 10^4^/µL	AST	21 U/L	CRP	6.44 mg/dL
Neu	64%	ALT	19 U/L	SAA	400.2 µg/mL
		ALP	85 U/L	ANA	<1:40
		BUN	7 mg/dL	RF	3.7 IU/ｍL
		Cr	0.46 mg/dL	anti‐CCP	2.1 U/mL

Abbreviations: Alb, albumin; ALP, alkaline phosphatase; ALT, alanine aminotransferase; ANA, antinuclear antibody; anti‐CCP, anti‐cyclic citrullinated peptide antibodies; AST, aspartate aminotransferase; BUN, blood urea nitrogen; Cl, serum chloride; Cr, creatinine; CRP, C‐reactive protein; Hb, hemoglobin; K, serum potassium; Na, serum sodium; Neu, neutrophils; PLT, platelets; RBC, red blood cell; RF, rheumatoid factor; SAA, serum amyloid A; T‐bil, total bilirubin; TP, total protein; WBC, white blood cell.

**FIGURE 1:**
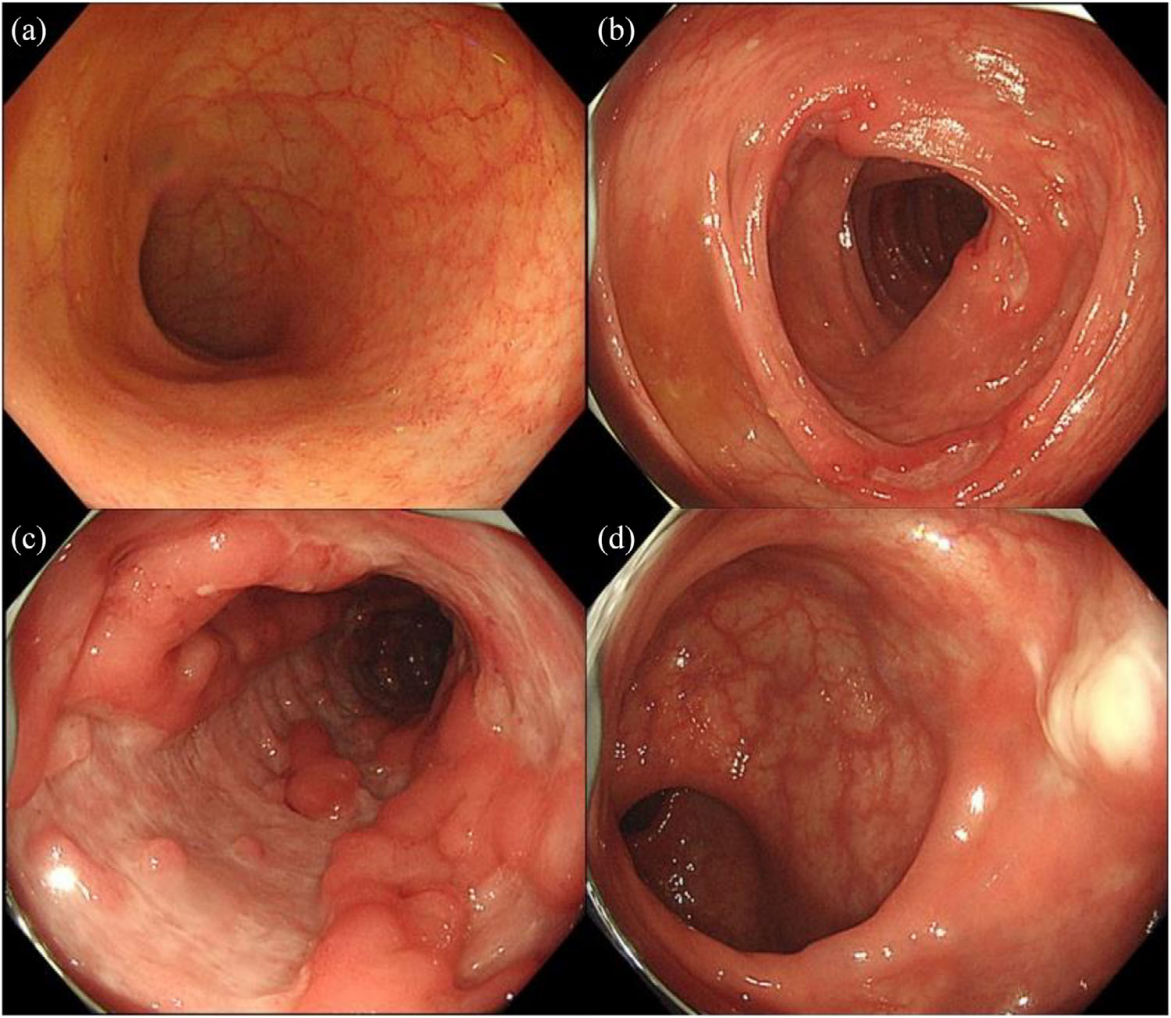
Colonoscopic findings of the case in May 2022. (a) There are no visible lesions in the terminal ileum. (b) Multiple round ulcers are present in the transverse colon. (c) Longitudinal ulcers with a cobblestone appearance are present in the descending colon. (d) A perianal abscess is present in the rectum.

**FIGURE 2:**
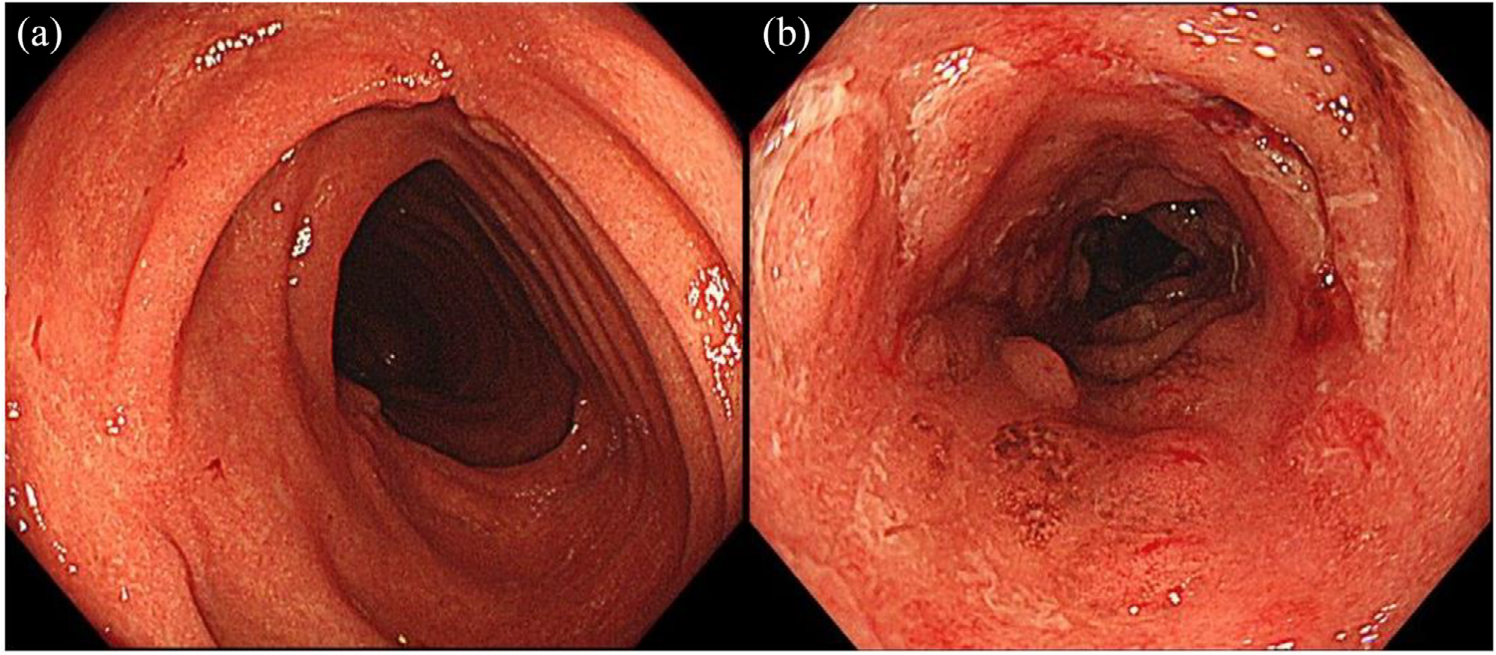
Colonoscopic findings, of the case in October 2022. Continuous, diffuse, and granular mucosal lesions are present throughout the entire colon, starting from the rectum. (a) Transverse colon. (b) Rectum.

Contrast‐enhanced computed tomography showed hyperenhancement of the bowel wall in the lower ileum and colon. A colonoscopy revealed multiple round ulcers scattered throughout the entire colon, longitudinal ulcers with an apparent cobblestone appearance in the descending colon and sigmoid colon, and a perianal abscess in the rectum. There were no visible lesions in the terminal ileum (Figure 1). Histologic examination of the biopsy specimens obtained from the margins of the ulcers showed inflammatory cell infiltration in the mucosa without any evidence of epithelioid cell granuloma or amyloidosis. Intestinal tuberculosis, cytomegalovirus enteritis, and other infectious diseases were excluded. Esophagogastroduodenoscopy with multiple biopsies did not show any findings compatible with CD. Based on the colonoscopic findings, we made a tentative diagnosis of CD and started intravenous infliximab, plus 3000 mg per day of 5‐aminosalicylic acid (ASA), and azathioprine (Figure 2).

**FIGURE 3:**
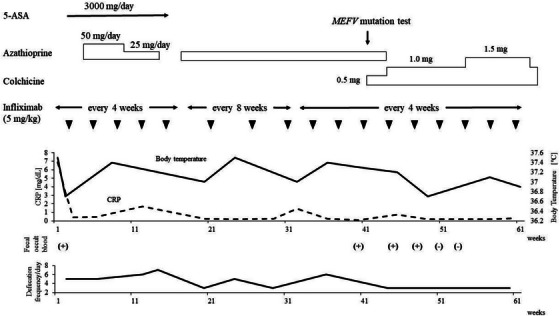
Clinical course of the case. 5‐ASA, 5‐aminosalicylic acid; CRP, C‐reactive protein.

Over the next 40 weeks, the patient experienced repeated fevers, episodes of abdominal pain, diarrhea, and increased CRP levels. We suspected 5‐ASA intolerance and discontinued the medication, but she continued to experience fevers. We next tried intensifying the dose of infliximab, stepping it up every 4 weeks, but this was ineffective. Follow‐up colonoscopy 5 months after the dose escalation showed drastic visual changes. There was now diffusely granular and friable mucosa with loss of vascularity throughout the entire large bowel, including the rectum (Figure 3). Biopsy specimens showed crypt distortion, crypt abscesses, and dense infiltration of inflammatory cells in the mucosa. The colonoscopic and histologic findings suggested a diagnosis of UC. Such drastic visual and tissue findings led us to search for other causes of intestinal inflammation. She did not have any symptoms suggestive of Bechet's disease including ileal ulcers, oral apthosis, uveitis, genital ulcers, and skin lesions.

Although she did not meet the major Tel‐Hashomer criteria, she had at least two of the minor and one of the supportive criteria. Thus, we suspected the possibility of FMF as a differential diagnosis of IBD‐U.

We checked the *MEFV* gene and found compound heterozygous variants of E148Q and L110P in exon 2. Based on these results, we added 0.5 mg of colchicine per day to her regimen, then increased the dose to 1.0–1.5 mg per day and discontinued azathioprine. The patient became afebrile, and her episodes of diarrhea resolved. The serum CRP levels have remained within normal limits on periodic follow‐ups.

## DISCUSSION

A hereditary periodic syndrome with autosomal recessive inheritance, FMF is characterized by periodic fever and serositis, including peritonitis and pleuritis. The MEFV gene is responsible for FMF. The *MEFV* gene codes for the pyrin protein, which is expressed in granulocytes, monocytes, dendritic cells, and synovial fibroblasts. Pyrin regulates caspase‐1 activation and suppresses the activation of interleukin (IL)‐1β in the inflammasome.^6^ Functional abnormality of the pyrin protein is the presumptive cause of FMF.

FMF is diagnosed based on the Tel‐Hashomer criteria^7^. The attack of FMF is categorized as being typical and incomplete on the basis of clinical findings.

Several reports have described the endoscopic findings of gastrointestinal lesions in patients with FMF. Since the initial description by Arasawa et al. in 2012, there has been an accumulation of case reports of FMF, describing initial misdiagnosis with treatment for CD or UC.^1^, ^2^, ^3^, ^4^, ^5^ In those case reports, however, the small and large intestinal lesions were heterogeneous and ranged widely with respect to colonoscopic features. Arasawa et al. reported a patient with FMF mimicking CD who had a circumferentially erythematous mucosa and erosions in the cecum, together with longitudinal erosions and polyposis‐like lesions in the right colon.^1^ In other reports, UC‐like colonoscopic findings have been characterized by loss of mucosal vascularity, multiple ulcers or erosions, and a friable colonic mucosa without rectal involvement.^3^, ^4^, ^5^ We observed serial changes in the intestinal involvement of FMF in our patient, and we found that the topical colonic inflammation altered from the typical appearance of CD to the typical appearance of UC during management with infliximab and ASA. While it remains obscure, changes in cytokine profiles by the treatments may have contributed to serial changes in colonic involvement in our patient.

Several mutations in *MEFV* have been reported as causative variants for FMF. The most common are M694V, M680I, V726A, and M694I in exon 10; followed by E148Q and E148V in exon 2.^8^ Major *MEFV* variants among Japanese patients are E148Q in exon 2 and M694I in exon 10.^9^ Examples of the phenotype‐genotype correlation are a Japanese patient with FMF and CD‐like lesions who had variant 910G>A in exon 2, and patients with UC‐like lesions who had different variants including 910G>A and R202Q in exon 2 and S503C in exon 5.^3^, ^4^, ^5^ The expression of *MEFV* is reportedly increased in the inflamed bowel mucosa of patients with IBD.^10^ Testing for *MEFV* gene mutation in patients with IBD could be a clue to the characteristics of ileocolonic involvement in FMF. Of interest, Asakura et al reported a Japanese patient with FMF who had CD‐like involvement and had *MEFV* variants identical to those seen in our patient. In this case report, there was a longitudinal ulcer in the terminal ileum and aphthous lesions in the proximal colon; UC‐like lesions were not evident.^2^ It thus seems possible that the ileocolonic involvement of FMF depends not only on the *MEFV* genotype.

In conclusion, we report on our experience with a patient with FMF who had endoscopic findings that drastically changed from those of CD to those of UC. These observations suggest that intestinal involvement in FMF can manifest as either typical CD or typical UC within the same patient and that FMF should be considered as a differential diagnosis of IBD, even when patients fulfill diagnostic criteria for IBD.

## CONFLICT OF INTEREST STATEMENT

Takayuki Matsumoto is a responsible and executive JGES member for DEN Open. The other authors declare no conflict of interest.

## ETHICS STATEMENT

Approval of the research protocol by an Institutional Reviewer Board. N/A.

Informed Consent. If not applicable, please write N/A.

Registry and the Registration. N/A.

Animal Studies. N/A.
